# Reactive oxygen species mediate TNF-α-induced inflammatory response in bone marrow mesenchymal cells

**DOI:** 10.22038/ijbms.2019.37893.9006

**Published:** 2019-11

**Authors:** Liuzhong Wu, Yaping Pan

**Affiliations:** 1 Department of Periodontics, School of Stomatology, China Medical University, Liaoning Province, China; 2 Department of Periodontics, Shenyang Stomatological Hospital, Shenyang, Liaoning Province, China

**Keywords:** BMSCs, Inflammation response, MAPK, NF-κB, Oxidative stress, TNF-α

## Abstract

**Objective(s):**

It is generally believed that the inflammatory response in bone marrow mesenchymal stem cells (BMSCs) transplantation leads to poor survival and unsatisfactory effects, and is mainly mediated by cytokines, including interleukin-1β (IL-1β), tumor necrosis factor-α (TNF-α). In this study, we explored the mechanisms underlying the TNF-α-induced inflammatory response in BMSCs.

**Materials and Methods:**

We treated BMSCs with TNF-α (1 and 10 ng/ml) for 5 days. The expression levels of key inflammatory mediators were evaluated by Real-time PCR. Intracellular ROS level was measured by using a 2, 7-dichlorofluorescein diacetate (DCF-DA).

**Results:**

We found that TNF-α treatment dramatically increased the expression levels of some key inflammatory mediators, including IL-6, IL-1β, IFN-γ and transforming growth factor β (TGF-β). Moreover, TNF-α induced intracellular oxidative stress by elevating intracellular reactive oxygen species (ROS) level, which is due to the increase of lipid peroxidation, the reduction of antioxidant Glutathione (GSH) levels and the inhibition of many antioxidant enzyme activities in BMSCs. Interestingly, 5 µM curcumin, a ROS scavenger, dramatically lowered the TNF-α-induced inflammatory response in BMSCs. In addition, TNF-α induced the activation of extracellular-signal-regulated kinases 1/2 (ERK1/2), c-Jun N-terminal kinases (JNK), p38 and their down-stream transcription factors nuclear factor kappa B (NF-κB) pathway.

**Conclusion:**

ROS mediated the TNF-α-induced inflammatory response via MAPK and NF-κB pathway, and may provide a novel strategy to prevent the inflammatory-dependent impairments in BMSCs.

## Introduction

Bone marrow mesenchymal stem cells (BMSCs) are characterized by their pluripotent differentiation and self-renewal capability, and now have been extensively applied in different fields, especially in BMSCs transplantation (1). Therefore, BMSCs transplantation therapy is a promising strategy for treating multiple injuries, such as spinal cord injury. However, due to the inflammatory response and oxidative stress after BMSCs transplantation (2, 3), BMSCs transplantation therapy results in poor survival and does not produce expected results in some investigations (4-6).

Inflammatory response in BMSCs transplantation is mainly mediated by cytokines, including interleukin-1β (IL-1β), tumor necrosis factor-α (TNF-α). TNF-α is a pro-inflammatory cytokine released by macrophages acute inflammation, and is responsible for the periodontal inflammation mediated bone loss (7). In TNF-α transgenic mice, the expression levels of osteoblast marker genes were down-regulated, which were also found in suppression of osteogenesis in C57BL/6 MSCs after treatment with TNF-α (8, 9). Nuclear factor κB (NF-κB) is a negative regulator of bone formation and is found to be activated by TNF-α (10). After activation, NF-κB translocates into nucleus and involved in the pathogenesis of osteolysis in inflammatory diseases. In addition, reactive oxygen species (ROS) also involves in the activation of many intracellular signaling pathways, such as mitogen-activated protein kinases (MAPKs) (11). The MAPKs have a large effect in the gene expression of a large number of proinflammatory cytokines (12, 13).

Despite the fact that TNF-α affects the bone formation has been reported previously, the underlying cellular and molecular mechanisms of TNF-α on BMSCs are largely unknown. In the present study, TNF-α-induced inflammatory response in BMSCs was investigated, and we found oxidative stress induced by ROS mediated this process *via* MAPK and NF-κB pathway. 

## Materials and Methods

***Cell culture and treatment***

BMSCs primary cells ( ScienCell, 7500) were cultured to P2 generation in hmsc-bm medium ( ScienCell, 7501), and P3 generation cells were incubated on 60 mm cell culture plate. The hmsc-bm medium contains 5 µM/l β-glycerophosphate, 50 ug/ml vitamin C, and 1×10^-8 ^mol/l dexamethasone. Cells were exposed to a final concentration of TNF-α (1, and 10 ng/ml) for 5 days and then washed before conducting the bioassays. The reason to choose 1, and 10 ng/ml is based on the preliminary data about different concentrations of TNF-α treatments (0.1, 1, 10, 50, 100 ng/ml) in BMSCs proliferation by alamarBlue assay, showing that 0.1 ng/ml TNF-α treatment almost has no effect, and 10ng/ml TNF-α treatment has similar effect with 50, 100 ng/ml TNF-α.

***Cell proliferation activity assay***

Cell proliferation activity was evaluated by the alamarBlue method (14). In brief, BMSCs (1×10^5^ cells/ml) seeded in 96-well plates were incubated with TNF-α (1, 10 ng/ml) for 5 day. After the treatment, the cells were incubated with 10 % alamarBlue solution (1 mg/ml) for 4 hr, and the plates were then read by the plate reader (Multiscan Ascent 354, Labsystem, Finland) at wavelengths of 540 and 620 nm, respectively, to determine cell proliferation activity.

***Real-time polymerase chain reaction (PCR) analysis***

Total RNA were extracted from BMSCs using the TRIZOL (Invitrogen, Grand Island, NY, USA) according to the manufacturer’s protocol. Total RNA (500 ng) was reverse-transcribed to cDNA and conducted RT-qPCR by using PrimeScript RT reagent kit (Takara Co, Japan), and PCR amplification was performed by SYBR Premix Ex Taq II kit (Perfect Real Time, Takara, Japan). Real-time PCR was performed by using ABI PRISM 7900HT Fast PCR System (Applied Biosystems) according to the manufacturer’s instructions. Primers for human genes were designed and synthesized by Takara Co (Dalian, China) as follows: *Il-6*: forward (AGC GCC TTC GGT CCA GTT GC) and reverse (TGC CAG TGC CTC TTT GCT GCT); *Il-1β*:forward (TGG CGG CAT CCA GCT ACG AA) and reverse (CCG GAG CGT GCA GTT CAG TGA); *Ifn-r*: forward (GAA ACG AGA TGA CTT CGA AAA GC) and reverse (GCT GCT GGC GAC AGT TCA); *Tgf-β*: forward (CAA GTA GAC ATT AAC GGG TTC AGT TC) and reverse (GGT CGG TTC ATG CCA TGA AT); β-actin: forward (TGG CAC CCA GCA CAA TGA A) and reverse (CTA AGT CAT AGT CCG CCT AGA AGC A ). The cycle threshold (Ct) was determined using the cycle at which the primary (fluorescent) signal crossed a user-defined threshold. Quantification was normalized by the Ct value of β-actin by using the 2^−ΔΔCt ^formula.

***Measurement of intracellular ROS level***

Intracellular ROS level was measured by using a 2, 7-dichlorofluorescein diacetate (DCF-DA) detection kit according to manufacture’s instruction. Briefly, cells were washed twice with PBS buffer and digested with 0.25% trypsin. Then the cells were resuspended and incubated with 10 µM DCF-DA at 37 ^°^C for 30 min. After staining, the DCF fluorescence was analyzed using a FACSCalibur cytometer (BD Biosciences). In this study, 5 µM curcumin was used to scavenge intracellular ROS level. 

***Measurement of intracellular lipid peroxidation, superoxide dismutase (SOD), reduced glutathione (GSH) and glutathione reductase (GR) activities***

BMSCs (1×10^5^ cells/ml) were treated with TNF-α (1, 10 ng/ml) for 5 day. After that, cells were scraped off the dishes with a silicon “policeman” and transferred into Eppendorf tubes. Cells were then lysed in ice-cold PBS by sonication followed by centrifugation at 15,000 g for 10 min at 4 ^°^C. The resulting supernatants were used immediately for the following measurements.

The lipid peroxidation was analyzed by measuring the levels of malondialdehyde (MDA) using MDA assay kit from Keygen Biotech, Co, Ltd. (Nanjing, Jiangsu, China). SOD, GR, and GSH assay kits (Keygen Biotech, Co, Ltd.) were also used to determine the SOD activity, GR activity and measure the cellular reduced GSH, respectively. 

***Preparation of protein extracts and Western blot assay***

The BMSCs were seeded in 10 cm plates with fresh medium, and then exposed to 1 and 10 ng/ml TNF-α for 5 days. RIPA lysis buffer was used to extract total protein and the protein concentration was determined by bicinchoninic acid (BCA) assay. SDS-PAGE was carried out in 10% gel with equal-loading amount of protein per lane. After electrophoresis, the proteins were transferred to polyvinylidene fluoride (PVDF) membranes. After blocking with 5% BSA for 1 hr, the membrane was incubated with a 1:1000 dilution of primary antibody in 5% BSA at 4 ^°^C for overnight. After incubation, the membrane was washed with TBST containing 0.1% Tween-20, then anti-mouse and anti-rabbit IgG (1:5000 dilution) were incubated with the PVDF membranes at room temperature for 1 hr. Signals were developed on X-ray film using an enhanced chemiluminescence system (Eastman Kodak Company, USA).

Primary antibodies of P-JNK (#9251), ERK1/2 (#9102), JNK (#9252), P-ERK1/2 (#9101), P38 (#9212) and P-P38 (#9211), IkBa (#4812), P-IkBa (#2859) were purchased from Cell Signaling Technology (Cell Signaling, Danvers, USA), NF-kB (C-20: sc-372), Nrf2 (H-300: sc-13032), heme oxygenase-1 (HO-1) (H-105: sc-10789), GST-glutathione-S-transferase 1/2 (GSTO1/2) (FL-241: sc-98560), β-actin (I-19: sc-1616), and corresponding secondary antibodies were all purchased from Santa Cruz Biotechnology (Santa Cruz, CA, USA).

***Statistical analysis***

All data represent the mean ±SD of three independent experiments. Differences were considered significant if *P<*0.05. Western blot band intensity was analyzed using imagine J and statistical analyses were conducted using SPSS software.

## Results

***TNF-α induced the changes of cell morphology and cell proliferation in BMSCs***

**Figure 1:**
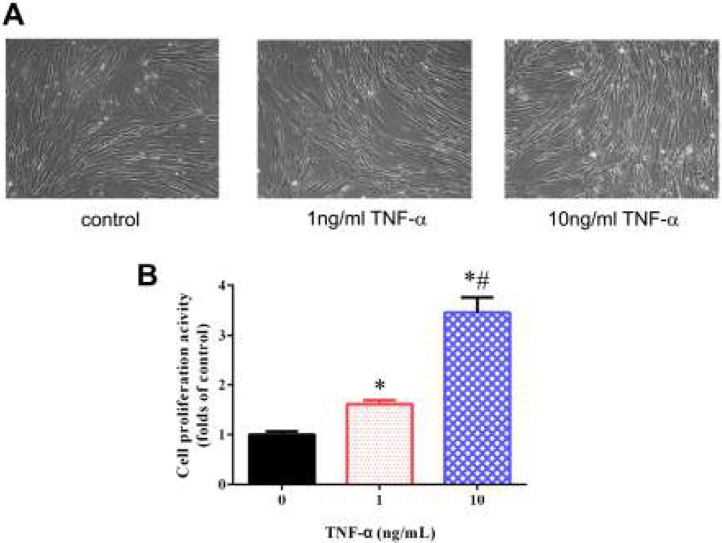
The effects of TNF-α on BMSCs morphology and cell proliferation

Incubation of BMSCs with TNF-α (1 and 10 ng/ml) for 5 days induced remarkable changes in cell morphology. Untreated BMSCs displayed a flat shape with round, triangular or spindle-shaped bodies and short processes, while TNF-α-treated BMSCs exhibited larger, more flattened or fibroblast-like morphology (Figure 1A). Here, we performed the alamarBlue assay to evaluate the effects of TNF-α on BMSCs proliferation. As shown in Figure 1B, TNF-α treatment significantly increased the proliferation activity of BMSCs in a does-dependent manner.

***TNF-α triggered inflammatory response in BMSCs***

**Figure 2:**
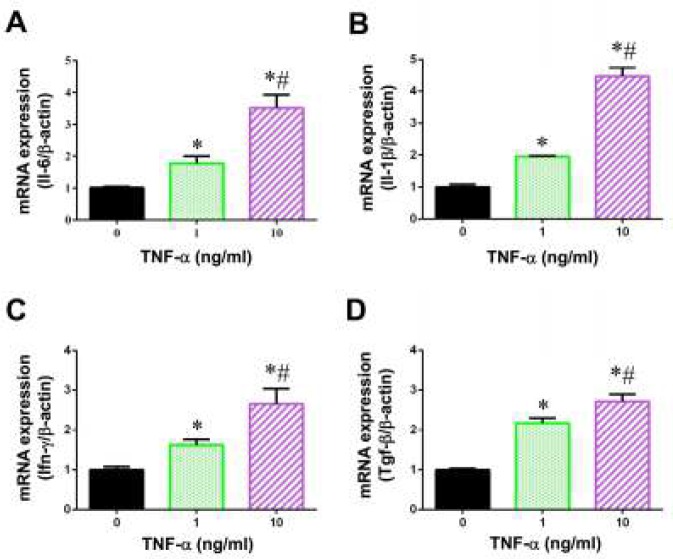
Effects of TNF-α on mRNA expression of inflammatory mediators in BMSCs

It is known that TNF-α treatment induced inflammatory response in BMSCs. To confirm this, we detected the expression levels of several key inflammatory mediators, including IL-6, IL-1β, IFN-γ and TGF-β by quantitative real-time PCR. We found that there was a quite similar change of gene expression profile of IL-6, IL-1β, IFN-γ and TGF-β after treating BMSCs with TNF-α for 5 days (Figure 2). Exposure of cells to 1 and 10 ng/ml TNF-α for 5 days increased 1.78- and 3.52-fold in the expression of IL-6, and 1.96- and 4.47-fold in the expression of IL-1β, respectively. Similarly, BMSCs with TNF-α resulted in a significant up-regulation in the mRNA expression of IFN-γ and TGF-β in a does-dependent manner. These results indicated that TNF-α induced cellular inflammatory response in BMSCs.

***TNF-α induces cellular oxidative stress in BMSCs***

**Figure 3:**
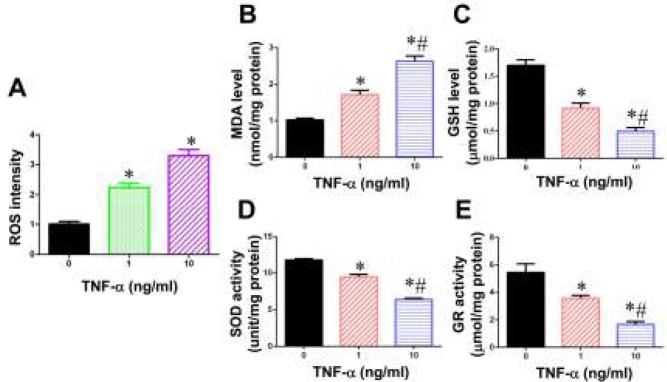
Effects of TNF-α on oxidative stress in BMSCs

To explore the mechanisms underlying the TNF-α-induced inflammatory response in BMSCs, we measured the intracellular ROS levels using DCFDA assay. As shown in Figure 3A, the ROS content was dramatically elevated to 2.2 fold and 3.3 fold after 1 and 10 ng/ml TNF-α treatment. This ROS increase may result from the imbalance of the redox system. Expectedly, comparing with the control group, the concentration of MDA (a common end product of lipid peroxidation) was increased about 71 % and 164 % following 1 and 10 ng/mL of TNF-α administration, respectively (Figure 3B), and the level of reduced GSH was markedly decreased by TNF-α (Figure 3C), reflecting the generation of lipid peroxidation and the imbalance of oxidative redox status. Moreover, the activities of SOD and GR, two well-known antioxidant enzymes, were found to be significantly lower in TNF-α treated BMSCs than control group (Figure 3D and 3F). Taken together, these changes indicated that TNF-α induced the imbalance of oxidants and antioxidants, and triggered an oxidative stress in BMSCs.

***ROS mediated the TNF-α-induced inflammatory response in BMSCs***

**Figure 4:**
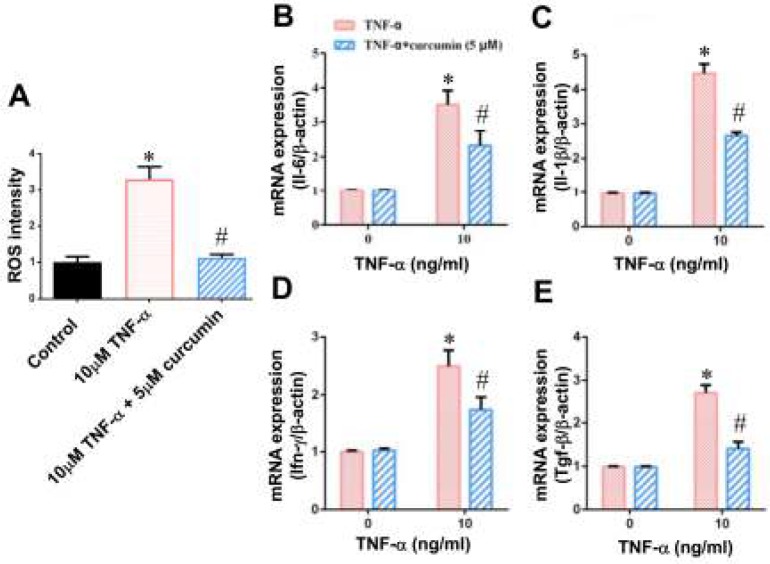
ROS scavenger lowered the TNF-α-induced inflammatory responses in BMSCs

To investigate whether the oxidative stress is involved in the TNF-α-induced inflammatory response in BMSCs, here we used ROS scavenger (5 µM curcumin) to lower the intracellular ROS level and checked the inflammatory response in BMSCs after treatment with 10 ng/ml TNF-α. We found that intracellular ROS level was dramatically decreased by 5 µM curcumin (Figure 4A). Next, we detected the expression levels of Il-6, Il-1β, Ifn-γ and Tgf-β after pre-incubation of BMSCs with 5 µM curcumin. As shown in Figure 4B, the increase in the mRNA levels of IL-6, IL-1β, IFN-γ and TGF-β induced by 10 ng/ml TNF-α was significantly decrease by 5 µM curcumin (Figure 4B), indicating that ROS mediated the TNF-α-induced inflammatory response in BMSCs. 

***MAPK and NF-κB pathway involved in TNF-α-induced inflammatory response in BMSCs***

**Figure 5:**
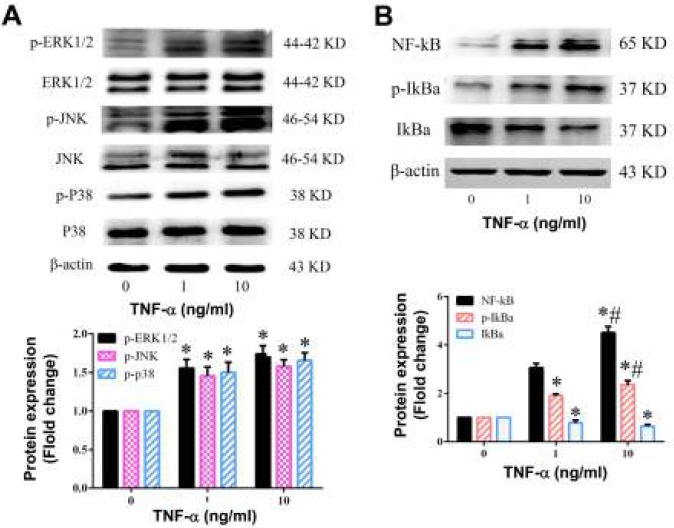
Effect of TNF-α on MAPK and NF-kB pathway in BMSCs

It is reported that MAPK pathway plays important roles in the regulation of several genes involving in immune and inflammatory responses through the regulation of transcription factors NF-κB and AP-1 (15). To investigate whether MAPK and NF-κB pathway involve in TNF-α-mediated inflammatory response in BMSCs, we examined the protein expression levels of genes related to the MAPK pathway, including JNK, ERK, and p38 using western blot. As shown in Figure 5A, administration of TNF-α resulted in a dose-dependent increase in the phosphorylation of ERK1/2, JNK and P38 MAPK, while the expression levels of ERK1/2, JNK and P38 MAPK have no change. These results indicated that MAPK pathway may involve in TNF-α-induced inflammatory process.

Since NF-κB is also an important redox-sensitive transcription factor, we postulated that TNF-α might activate NF-κB signaling. In the canonical NF-κB pathway, NF-κB activation depends on IκBα phosphorylation and degradation. We thus examined the effect of TNF-α on NF-κB pathway in BMSCs. We found that TNF-α significantly increased NF-κB protein expression; TNF-α also enhanced IκB-α phosphorylation and decreased the levels of IκB-α (Figure 5B), indicating that TNF-α exposure activated NF-κB signaling pathway.

(A) Microscopic analysis of BMSCs were viewed under Nikon TE 2000U microscope at x200 magnification. (B) Cell proliferation activity was determined by alamarBlue method and the percent of reduced alamarBlue of different treatment groups was calculated and finally expressed as folds of control cells. Results were expressed as mean±SD, n=3. * denoted *P<*0.05 versus control; # denoted *P<*0.05 versus corresponding TNF-α treated for 1 ng/ml, respectively

BMSCs: bone marrow mesenchymal stem cells; TNF-α: tumor necrosis factor-α

Relative mRNA levels of IL-6 (A), IL-1β (B), IFN-γ (C) and TGF-β (D) were normalized to β-actin, and finally expressed as folds of control cells. Differences were evaluated using the one-way ANOVA on ranks test. Results were expressed as mean±SD (n=4), and three independent experiments were carried out. *denoted *P<*0.05 compared with control, #denoted *P<*0.05 compared with 1 ng/ml TNF-α treatment group

BMSCs: bone marrow mesenchymal stem cells; IL-6: interleukin-6; IL-1β:interleukin-1β; IFN-γ: Interferon-γ; TGF-β: transforming growth factor β; TNF-α: tumor necrosis factor-α

(A) Intracellular ROS level was measured using DCFDA assay. (B) MDA level, (C) reduced GSH, (D) SOD activity and (E) GR activity in different groups. Results were expressed as mean±SD, n

=4. * denoted *P<*0.05 versus control; # denoted *P<*0.05 versus corresponding TNF-α treated for 1 ng/ml, respectively

BMSCs: bone marrow mesenchymal stem cells; MDA:malondialdehyde; GSH: glutathione; SOD: superoxide dismutase; GR: glutathione reductase; TNF-α: tumor necrosis factor-α; ROS: reactive oxygen species

(A) ROS scavenger lowered intracellular ROS level in TNF-α treated group. (B) the mRNA expression levels of IL-6, IL-1β, IFN-γ and TGF-β after pre-incubation of BMSCs with 5 µM curcumin are significantly decreased. Results were expressed as mean±SD, n=4. * denoted *P<*0.05 versus control

BMSCs: bone marrow mesenchymal stem cells; ROS: reactive oxygen species; IL-6: interleukin-6; IL-1β:interleukin-1β; IFN-γ: Interferon-γ; TGF-β: transforming growth factor β; TNF-α: tumor necrosis factor-α

(A) Expressions of ERK1/2, JNK and P38 and (B) expressions of NF-kB, p-IkBa, IkBa in BMSCs were assessed by western blotting. β-actin was blotted as the loading control. Data were representative of three independent experiments. Results were expressed as mean±SD (n=4), and two such independent experiments were carried out. *denoted *P<*0.05 compared with control mice

BMSCs: bone marrow mesenchymal stem cells; TNF-α: tumor necrosis factor-α; MAPK:mitogen-activated protein kinase; ERK1/2:extracellular signal-regulated kinase1/2; JNK: c-Jun N-terminal kinase

**Figure 6:**
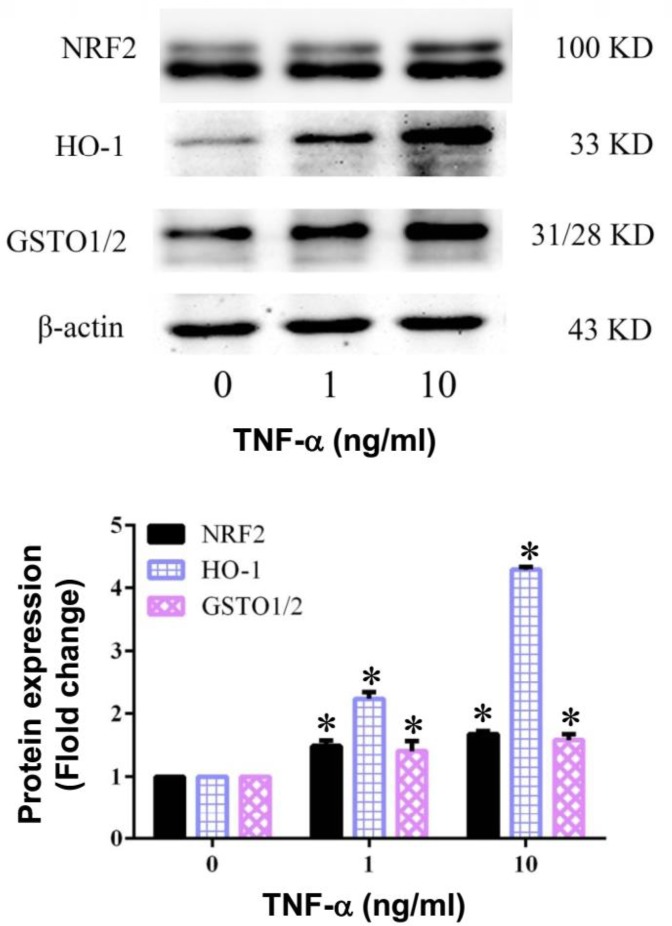
Effect of TNF-α on Nrf2 pathway in BMSCs

Expressions of NRF2 and its down-stream target HO-1 and GSTO1/2 in BMSCs were assessed by Western blotting. β-actin was blotted as the loading control. Data were representative of three independent experiments. Results were expressed as mean±SD (n=4), and two such independent experiments were carried out. * denoted *P<*0.05 compared with control mice

BMSCs: bone marrow mesenchymal stem cells; GSTO1/2: Glutathione S-transferase mega 1/2; Nrf2:Nuclear factor erythroid 2–related factor 2; TNF-α: tumor necrosis factor-α

***TNF-α activates NRF2 pathway in BMSCs***

In this study, we first observed the expression of NRF2 protein in BMSCs. As shown in Figure 6, TNF-α treatment showed a significant increase of NRF2 protein in BMSCs. Here, the expression of HO-1 and GSTO1/2, two well-known targets of Nrf2 signaling pathway, were also detected after TNF-α treatment. Both the HO-1 and GSTO1/2 proteins expression in BMSCs were significantly increased after 1 and 10 ng/ml TNF-α treatment. It is therefore suspected that the stimulation of Nrf2 signaling pathway may also involve in the TNF-α-induced inflammatory response on BMSCs.

## Discussion

This study explored the effect of TNF-α on cell proliferation and inflammatory responses in BMSCs as well as the underlying mechanisms. Although many experiments have observed that TNF-α could impair differentiation of MSCs, but the underlying mechanisms remain poorly understood. Here, we found TNF-α significantly increased intracellular ROS level, and ROS scavenger dramatically lowered the TNF-α-induced inflammatory response in BMSCs, indicating that oxidative stress mediated TNF-α-induced inflammatory response, probably *via *activation of MAPK and NF-κB pathway. This study provides a better understanding of the effects of TNF-α on BMSCs. 

Inflammation response is a series of cellular and molecular responses that defend the body from infections or other impairments (16, 17). It has been demonstrated that TNF-α involved in the inflammation response in the BMSCs transplantation (18). TNF-α is reported to promote the inflammatory cell infiltration by leukocyte adhesion molecules on endothelial cells and activate phagocytes killing mechanisms (19). IL-6 works as a messenger cytokine in the expression of C-reactive protein, fibrinogen, and plasminogen activator inhibitor-1 and accelerates oxygen radical production . Its levels positively correlate with higher all-cause mortality (20). Some reports found increased mRNA expression of IL-6 in MSCs stimulated with TNF-α and IL-1β, suggesting pro-inflammatory cytokines can attract MSCs to sites of inflammation (21). In line with this observation, we found TNF-α treatment increased the mRNA expression of IL-6, IL-1β, IFN-γ and TGF-β in BMSCs, and therefore verified the robust pro-inflammatory responses occurred after TNF-α treatment.

Previous studies have showed that TNF-α induces the generation of cellular ROS in different tissues through stimulating ROS biogenesis (22) and enhancing the transcription of various NADPH oxidase components, or triggering NADPH oxidase activation (23). In addition, the decline of enzymatic antioxidant system mainly resulted in redundant ROS. In our experiments, we found that TNF-α significantly increased the level of intracellular ROS, which was resulted from the imbalance of oxidants and antioxidants, such as increase in MDA level, disturbed GSH balance and decrease in SOD and GR activities. More importantly, ROS scavenger dramatically lowered the TNF-α-induced inflammatory response in BMSCs, indicating that ROS mediated TNF-α-induced inflammatory response in BMSCs.

## Conclusion

Our results demonstrate that TNF-α promoted cell proliferation and induced inflammatory response by altering the expression of pro-inflammatory cytokines in BMSCs. Moreover, TNF-α induced oxidative stress via activating MAPK, NF-κB and NRF2 pathway in BMSCs, but which is main pathway need further study. These finding shed a new light on the study of molecular mechanism of inflammation-induced dysfunction of BMSCs.
